# Combined Laparoscopic–Robotic Partial Nephrectomy: A Comparative Analysis of Technical Efficiency and Safety

**DOI:** 10.3390/jcm14248693

**Published:** 2025-12-08

**Authors:** Irfan Safak Barlas, Mehmet Yilmaz, Halil Cagri Aybal, Mehmet Duvarci, Selcuk Guven, Lutfi Tunc

**Affiliations:** 1 Department of Urology, Acibadem Ankara Hospital, 06450 Ankara, Turkey; lutfi.tunc@acibadem.com; 2 Department of Urology, Mediclin Kraichgau Klinik, 74906 Bad Rappenau, Germany; mehmet.yilmaz@mediclin.de; 3 Department of Urology, Gulhane Training and Research Hospital, 06010 Ankara, Turkey; halilcagri.aybal@saglik.gov.tr; 4 Department of Urology, Dr. Abdurrahman Yurtaslan Ankara Oncology Training and Research Hospital, 06200 Ankara, Turkey; mehmet.duvarci@sbu.edu.tr; 5 Department of Urology, Meram School of Medicine, Necmettin Erbakan University, 42090 Konya, Turkey; sguven@erbakan.edu.tr

**Keywords:** small renal mass, renal cell carcinoma, surgical technique, robot-assisted laparoscopic surgery, laparoscopic surgery

## Abstract

**Background/Objectives:** We aimed to evaluate the feasibility and safety of a combined approach to partial nephrectomy, which involves laparoscopic dissection for kidney as well as renal hilum mobilization, followed by robotic assistance for tumor resection and intracorporeal suturing, integrating the technical advantages of both laparoscopic and robotic surgery. **Methods:** We retrospectively analyzed 99 patients with clinical stage 1 renal tumors who underwent laparoscopic (LPN, n = 31), robot-assisted (RAPN, n = 16), or combined partial nephrectomy (CPN, n = 52) between 2016 and 2024. CPN involved laparoscopic mobilization of the kidney and renal hilum, followed by robotic tumor excision and intracorporeal suturing. Perioperative and postoperative outcomes were compared across groups. **Results:** Comparative analysis of the demographic characteristics of patients who underwent LPN, RAPN and CPN revealed no significant differences. The mean operative time (OT) was 126.75 ± 25.28 min for CPN, 121.9 ± 9.5 min for LPN (*p* = 0.014), and 155.5 ± 18.03 min for RAPN (*p* < 0.001). The median warm ischemia time (WIT) was 20.0 min (10.0–26.0) for CPN, which is comparable to RAPN at 18.5 min (14.0–23.0) (*p* = 0.158), but it was significantly longer for LPN at 23.0 min (18.0–28.0) (*p* < 0.001). The estimated blood loss (EBL) was 120.0 mL (50.0–350.0) for CPN, which is similar to RAPN at 110.0 mL (50.0–300.0) (*p* = 0.158), while it was higher for LPN at 180.0 mL (100.0–250.0) (*p* < 0.001). No major intraoperative or postoperative complications classified as Clavien–Dindo grade ≥3 were observed in any group. **Conclusions:** CPN is a feasible and safe approach for clinical stage 1 renal tumors, combining the efficiency of laparoscopy with the precision of robotics. Compared with LPN and RAPN, CPN showed comparable early oncological and functional results and had shorter operative duration and improved perioperative parameters.

## 1. Introduction

Renal cell carcinoma (RCC) is the third most common urological malignancy and accounts for approximately 3 percent of all adult cancers worldwide [1]. The widespread adoption of advanced imaging modalities has led to increased incidental detection of small renal masses, most of which are localized and suitable for nephron-sparing surgery [2,3]. In this clinical context, partial nephrectomy (PN) has become the standard treatment option for patients with clinical stage 1 RCC. Beyond providing oncological control comparable to radical nephrectomy (RN), the fundamental principle of PN is to increase the amount of preserved functional renal parenchyma, thereby minimizing postoperative loss of kidney function. Accordingly, the intraoperative principles of nephron-sparing surgery focus on protecting functional renal units, with warm ischemia time (WIT) serving as a parameter used intraoperatively to anticipate the extent of procedure-related damage. In line with this objective, various surgical modalities and techniques have been developed to further enhance nephron preservation and minimize damage to functional renal units [4].

Minimally invasive surgical approaches, particularly laparoscopic partial nephrectomy (LPN), have demonstrated favorable perioperative outcomes with reduced morbidity and shorter hospital stays when compared to open surgery. However, despite these advantages, LPN remains technically demanding, especially during tumor excision and intracorporeal renorrhaphy. These challenges may result in prolonged WIT and increased procedural complexity, potentially affecting renal functional outcomes [3,5]. As a result, efforts have been made to overcome these limitations through the integration of technological innovations.

Robot-assisted partial nephrectomy (RAPN) has emerged as a valuable advancement in minimally invasive urologic surgery, offering enhanced dexterity, improved visualization, and greater precision during tumor excision and renorrhaphy [6,7]. Although RAPN has demonstrated reductions in ischemia time and improvements in surgical ergonomics during critical phases of partial nephrectomy, it offers limited benefit during early steps such as kidney and hilar mobilization, as well as tumor localization, where laparoscopy provides the advantage of tactile feedback and faster dissection [6,7,8,9].

In practical clinical scenarios, particularly when laparoscopic dissection presents intraoperative challenges for tumor resection or renal repair, a stepwise hybrid approach is occasionally employed. This combined laparoscopic-robotic partial nephrectomy (CPN) involves laparoscopic mobilization of the kidney and renal hilum, followed by robotic assistance for tumor excision and intracorporeal suturing. The rationale for this approach lies in integrating the speed and accessibility of laparoscopy with the technical precision and control provided by robotic systems. Although this method is increasingly utilized in high-volume centers, data evaluating its efficacy and safety remain limited. The present study aims to assess the feasibility, perioperative outcomes, and complication rates of CPN in patients with clinical stage 1 renal tumors by comparing it to conventional LPN and RAPN techniques. We hypothesized that the CPN approach would offer a favorable balance, combining the technical efficiency of laparoscopy with the reconstructive advantages of robotic surgery, while maintaining surgical safety and effectiveness.

## 2. Material and Method

### 2.1. Study Design and Data Collection

Between June 2016 and May 2024, a total of 107 patients who underwent LPN, RAPN and CPN at the Department of Urology, Acibadem Ankara Hospital, were evaluated. The data were collected retrospectively after the Acibadem University’s Ethics Committee’s approval (2024-10/440, 18 July 2024).

**Figure 1:**
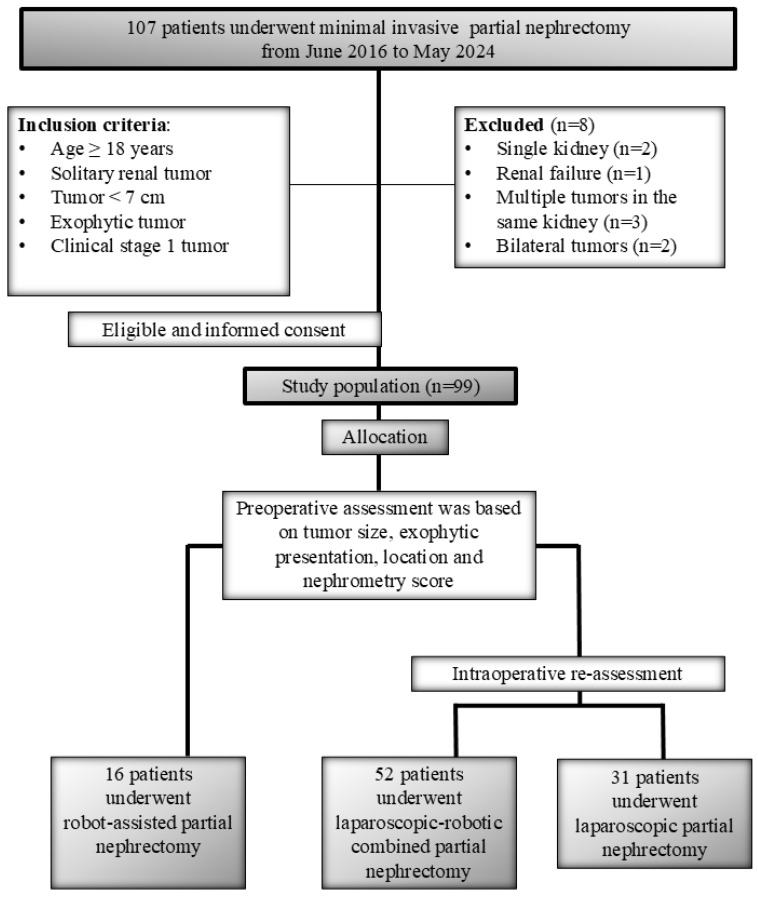
Diagram of Patient Selection and Allocation for Minimally Invasive Partial Nephrectomy.

The clinical data of these patients were recorded in the hospital’s electronic medical database. The preoperative evaluation included abdominal imaging using contrast-enhanced computed tomography or magnetic resonance imaging. The study included patients aged over 18 with solitary renal tumors that were technically feasible for resection, with a maximum tumor diameter of less than 7 cm, classified as clinical stage 1 tumors according to TNM (Tumor, Node, Metastasis) staging. Exclusion criteria were patients with tumor thrombus, metastatic disease, lymph nodes thought to have metastasis, endophytic or centrally located tumors, multiple tumors, a history of previous abdominal surgery, incomplete data, or unavailability of surgical video records. The diagram of patient selection presents the information related to excluded patients, and the allocation of patients according to the surgical approach (Figure 1).

Preoperatively, the RENAL nephrometry score [10] was used to assess tumor characteristics. We collected demographic data such as sex, age (year), comorbidities, and body mass index (BMI). Preoperative data included estimated glomerular filtration rate (eGFR) (mL/min/1.73 m^2^), serum creatinine (Cre) (mg/dL), and hemoglobin (Hb) (g/dL) levels, along with radiographic measurements of tumor size (mm), side and localization. Perioperative variables included total operative time (OT) (min), kidney dissection time (min), vascular loop placement time (min), tumor margin marking time (min), tumor resection time (min), parenchymal reconstruction time (min), warm ischemia time (WIT) (min), and estimated blood loss (EBL) (mL). Postoperative values of Hb, Cre, and eGFR were measured on postoperative day 2 and compared with preoperative values. Hospitalization time (days), tumor pathology, surgical margin status, complications (Clavien-Dindo) [11], and transfusion requirements were also recorded. TNM 2017 was used for clinical staging [12].

### 2.2. Laparoscopic-Robotic Combined Partial Nephrectomy Surgical Technique

The procedure was initiated with a transperitoneal laparoscopic approach. The patients were then repositioned into a lateral decubitus position. Pneumoperitoneum was established using carbon dioxide insufflation. The first incision, approximately 1 cm in size, was made superior to the umbilicus. The second 12 mm trocar was placed 8 cm lateral to the first. The third 12 mm trocar was inserted in a mirrored fashion to the second.

**Figure 2:**
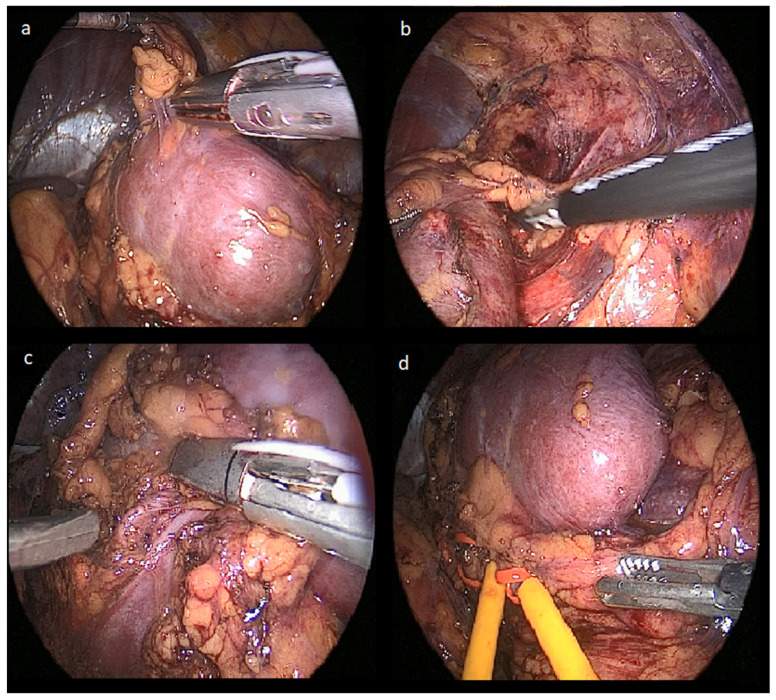
Images Demonstrating the Steps of the Laparoscopic Approach in the Initial Part of Combined Partial Nephrectomy. (**a**). Using a laparoscopic approach, the kidneys are shown dissected from the surrounding tissues and mobilized (**b**). Gerota’s fascia is opened to localize the tumor, and the mass is carefully dissected from the surrounding perirenal fat, allowing clear visualization of the tumor (**c**). After dissecting the renal hilum, the renal artery and vein were identified (**d**). A loop-shaped vascular tape was loosely applied around the renal artery and vein separately, for renal pedicle control.

We mobilized the descending colon and medially displaced it for patients with left-sided kidney tumors. In cases involving right-sided kidney tumors, a modified descending nephrectomy (Tunc Technique) [13] was performed, allowing access to the upper pole and renal hilum through dissection of the hepatorenal recess. In both kidneys, the upper pole was exposed, and the kidneys were dissected from the surrounding tissues and mobilized (Figure 2a). Gerota’s fascia was subsequently opened to localize the tumor. The mass was dissected from the surrounding perirenal fat, allowing clear visualization of the tumor (Figure 2b). The kidney was then elevated to expose the renal hilum (Figure 2c). After dissecting the renal hilum, the renal artery and vein were identified, and a loop-shaped vascular tape was loosely applied around the renal hilum for renal pedicle control (Figure 2d). All these steps were performed completely laparoscopically using laparoscopic instruments.

All patients with low or moderate nephrometry complexity based on preoperative RENAL scoring were initially planned for a laparoscopic approach. After laparoscopic mobilization and dissection of the mass, an intraoperative reassessment was performed. In cases where the intraoperative RENAL score was recalculated and found to be higher than the preoperative score, conversion to CPN was considered. This decision was taken particularly when intraoperative findings differed from preoperative imaging, such as a more prominent endophytic component than anticipated, tumor location very close to the renal hilum, or adhesions that made safe dissection difficult, or when tumor size or location caused ergonomic difficulties during dissection. In the presence of these challenges, a decision was made to convert to CPN to optimize tumor control and renal reconstruction. A robot-assisted laparoscopic procedure was then performed using the Da Vinci^®^ Surgical System (Intuitive Surgical, Inc., Sunnyvale, CA, USA) with a standard three-arm configuration. The 12 mm laparoscopic trocars were replaced with 8 mm robotic trocars through the same incisions, and the fascial openings were narrowed using sutures to prevent gas leakage. The robotic trocars were fixed in place with the same sutures. A 12 mm assistant trocar was placed in the plane between the camera port and the inferior robotic arm.

**Figure 3:**
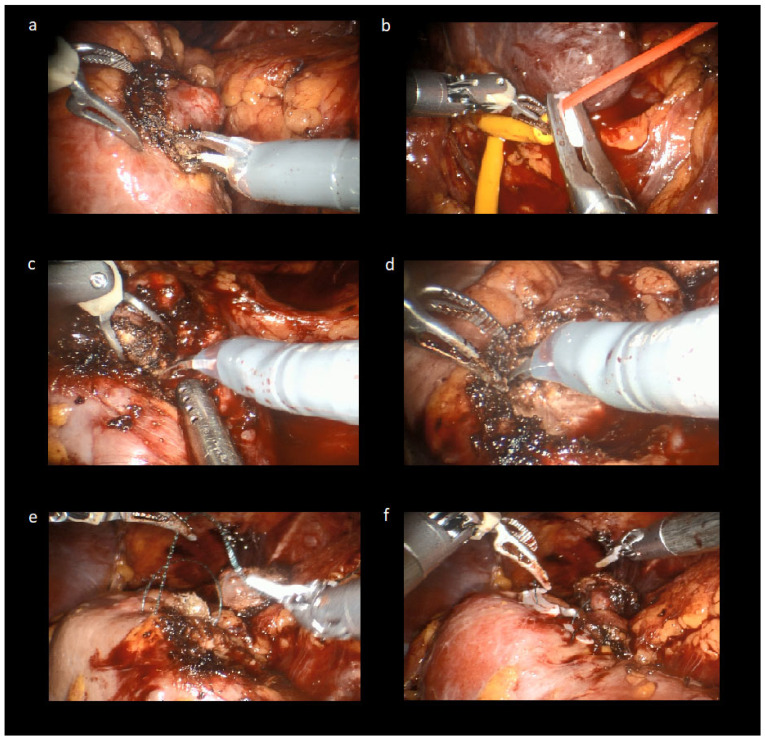
Images Demonstrating the Steps of the Robotic Approach in the Second Part of Combined Partial Nephrectomy. (**a**). Initially, in the robotic approach, the tumor margins were marked approximately 5 mm outside the tumor using robotic scissors with monopolar electrocautery, carefully separating the mass from the surrounding normal renal parenchyma (**b**). The renal hilum was tourniqued using the previously placed vascular loops to occlude the renal pedicle (**c**). Tumor resection was performed with robotic scissors and electrocautery (**d**). Deep resection was performed at the base of the tumor bed within the parenchyma to ensure complete excision (**e**,**f**). Renal parenchymal reconstruction was carried out in two layers where the first layer involved closing the tumor bed and the second layer was focused on parenchymal repair.

After docking the robot, approximately 5 mm outside the tumor margins were marked using robotic scissors with monopolar electrocautery, carefully separating the mass from the surrounding normal renal parenchyma (Figure 3a). The renal hilum was tourniqued using the previously placed vascular loops to occlude the renal pedicle (Figure 3b), and tumor resection was performed with robotic scissors and electrocautery (Figure 3c). The depth of the endophytic component was cognitively estimated based on preoperative imaging, and following tumor resection, deep resection was performed at the base of the tumor bed within the parenchyma to ensure complete excision (Figure 3d). Then, we carried out renal parenchymal reconstruction in two layers, with the first layer involving the closure of the tumor bed and the second layer focusing on parenchymal repair (Figure 3e,f). After the repair, the vascular tourniquet was cut and removed immediately to minimize WIT.

For patients with low nephrometry scores, where tumor resection and renal parenchymal repair could be easily performed laparoscopically based on tumor characteristics, the subsequent steps were executed laparoscopically, and the procedure was completed as an LPN. In these cases, all steps were identical to those in CPN, the tumor margins were marked using laparoscopic scissors with monopolar electrocautery (Covidien, Mansfield, MA, USA), the renal hilum was occluded by tourniquing, and tumor resection was performed with laparoscopic scissors and renal parenchymal reconstruction was carried out laparoscopically.

In the preoperative evaluation, patients with high nephrometry scores where tumor resection and renal parenchymal repair could not be easily performed laparoscopically due to tumor characteristics, all steps were executed using a robot-assisted laparoscopic approach from the outset, and the procedure was completed as a RAPN. In these cases, all steps were performed using the same techniques and order as in the other two procedures, following a robot-assisted laparoscopic approach.

### 2.3. Statistical Analysis

Statistical analyses were performed utilizing SPSS version 22.0. The Shapiro–Wilk and Kolmogorov–Smirnov tests were used to evaluate the normality of continuous variables. We presented normally distributed continuous variables as mean and standard deviation, while non-normally distributed variables were reported as median and interquartile range. The Mann–Whitney U test and independent samples t-test were used to compare continuous variables. The Pearson chi-squared test and Fisher’s exact test were utilized to compare categorical variables. A Spearman or Pearson correlation analysis was conducted to examine potential correlations. Propensity score-matched analysis (PSM) was conducted to reduce the effects of bias using logistic regression with a caliper value of 0.01, incorporating tumor size. A 1:1:1 CPN: LPN: RaPN group matching was done using PSM. The criterion for statistical significance was set at *p* < 0.05. In the comparison of the two groups, statistical significance was set at *p* < 0.016.

## 3. Results

The preoperative characteristics of patients who underwent LPN, RAPN, and CPN are presented in Table 1.

The preoperatively calculated RENAL nephrometry score and tumor size, along with the intraoperatively recalculated RENAL nephrometry score, are presented in the comparison of patients underwent LPN, RAPN, and CPN (Table 2). Following adjustment with propensity score matching, the distribution of preoperative RENAL nephrometry scores and tumor size across the matched groups is summarized in Table 3.

The comparison of perioperative data and the time required for each intraoperative step among the groups are presented in Table 4. The median tourniquet placement time was 3.7 min (2.5–6.5) in LPN and 4.0 min (3.0–5.0) in CPN (*p* = 0.820), both performed using laparoscopic instruments. In contrast, in RAPN, where tourniquet placement was performed using robotic arms, the median duration was significantly longer at 7.5 min (5.0–11.5) compared to both LPN (*p* < 0.001) and CPN (*p* < 0.001). Perioperative parameters included in Table 4 were also examined after propensity score matching, and the matched-group comparisons are presented in Table 5.

Additionally, the median tumor margin marking time, performed using monopolar energy before tumor resection, was 22.0 min (12.0–29.0) in LPN, where laparoscopic instruments were used. In comparison, this step was significantly shorter in CPN at 10.25 min (4.0–24.0) (*p* < 0.001) and in RAPN at 11.5 min (5.0–15.0) (*p* < 0.001), both performed using robotic arms. There was no significant difference between CPN and RAPN (*p* = 0.805).

The postoperative characteristics and data of patients who underwent different surgical techniques were analyzed, and the groups were compared (Table 6). In particular, the pathological examination of the specimen from one patient in CPN group with clear cell RCC Fuhrman grade 2 revealed tumor contact with the surgical margin in one area; however, no tumor was detected in the deep resection. Furthermore, no recurrence was observed during the two-year follow-up. Another patient in CPN group, clinically staged as T3a due to perirenal tissue invasion in the postoperative pathology with clear cell RCC Fuhrman grade 3, was found to have a negative surgical margin but experienced recurrence during follow-up. In another patient in LPN group, whose postoperative pathology revealed clear cell RCC Fuhrman grade 4, a negative surgical margin, and a clinical stage of T1b, a de novo tumor developed in a different location in the same kidney during follow-up.

Following the laparoscopic approach and before initiating the robotic approach, no complications or difficulties were encountered during trocar Exchange in CPN group. None of the patients required conversion to laparotomy or RN and no major or hemorrhagic complications were observed. Furthermore, none of the patients developed acute renal failure following the operation.

## 4. Discussion

In our study, we presented our combined partial nephrectomy method, in which we employed both laparoscopic and robotic approaches in the surgical treatment of clinical stage 1 renal tumors. This hybrid strategy enabled the use of each modality in the steps where it offers technical advantages. Compared with LPN and RAPN, CPN was found to have comparable surgical margin status, early oncological outcomes, and early postoperative renal functional results, without introducing additional limitations associated with either approach. Given these comparable outcomes, CPN may represent a feasible alternative for selected patients who do not require open partial nephrectomy.

Although WIT has traditionally been considered a key determinant of postoperative renal function [14,15], contemporary evidence increasingly suggests that long-term renal outcomes are influenced more profoundly by the residual renal parenchyma amount and by patients’ comorbidities, as well as by the type of ischemia applied, rather than by small differences in ischemia duration alone [16]. In this context, the ergonomic advantages of robotic assistance through articulated instruments, enhanced dexterity, and three-dimensional magnified visualization gain relevance during tumor resection and parenchymal repair [7,14,15,17]. In our cohort, the phases performed robotically in RAPN and CPN were associated with shorter WIT (18.5 and 20.0 min, respectively) compared with LPN (23.0 min). While these numerical differences may not independently translate into measurable long-term functional benefits, they likely reflect a more controlled and precise resection and repair process, enabling maximal preservation of healthy parenchyma and minimizing inadvertent tissue trauma. Thus, rather than interpreting shorter WIT as a direct clinical advantage, these findings may instead highlight the technical refinement afforded by robotic assistance during the most critical steps of nephron sparing surgery.

Although RAPN reduces WIT due to technical advantages in suturing during tumor resection and renal parenchymal repair, it does not significantly shorten the overall OT, as the benefits gained in these specific phases are not reflected throughout the entire procedure [8,14,18,19]. While robotic surgery allows for faster tumor resection and suturing compared to LPN, it has not demonstrated superiority in other stages of the surgery. Furthermore, its slower pace compared to laparoscopic approach limits any substantial reduction in OT [6,8]. Prolonged OT is associated with a higher risk of perioperative complications [20].

In this study, however, the mean OT was significantly lower with CPN (126.7 min) compared to both LPN (136.8 min) and RAPN (155.5 min), likely due to the strategic integration of laparoscopic and robotic approaches during the phases where each offered the greatest efficiency. Although the literature presents inconsistent findings regarding OT, with studies favoring RAPN or LPN and reporting minimal differences [14,17], our results indicate that CPN achieved the shortest OT reported to date among partial nephrectomy procedures, suggesting an operative advantage of this combined technique.

Intraoperative EBL is a key surgical parameter, as excessive bleeding may obscure the operative field, increase complication risk, and necessitate conversion to open surgery [21]. It has also been associated with higher rates of positive surgical margins [22]. In our study, the median EBL was 120 mL for CPN, 180 mL for LPN, and 110 mL for RAPN. Our overall complication rate in the CPN group was low, and none of the patients experienced major complications of grade 3 or higher, nor was there a need to convert to open surgery or RN. These findings are notable given that previous studies have reported higher EBL and complication rates [14,17]. The lower EBL observed with CPN compared to the purely laparoscopic approach may be attributed to the advantages of robotic assistance during the tumor resection and renorrhaphy phases, while the initial laparoscopic phase allows tactile feedback that facilitates tumor localization and preparation of the kidney for resection, potentially reducing the risk of injury to adjacent organs [23].

Achieving a negative surgical margin is a key parameter when comparing partial nephrectomy methods. In our series, only one patient’s specimen demonstrated contact with the surgical margin, and no tumor was identified in the deep resection in any case. These findings suggest that the procedures might be feasible with secure tumor resection at the time of surgery. However, while the absence of residual tumor supports the technical safety of the approaches used, it remains too early to draw firm conclusions regarding oncological results.

During renal hilum dissection performed as part of CPN, the laparoscopic approach was preferred because it allows for a shorter operative time and provides tactile feedback, but it offered no additional benefit beyond reducing OT, as no differences were observed in the number of intraoperative complications or in EBL. Although no complications occurred, the increasing adoption of CPN may raise concerns regarding the potential for complications and lead surgeons to avoid attempts to shorten the duration of this step. With ongoing advancements in robotic systems that offer improved tactile feedback, performing renal hilum dissection robotically in a more controlled manner may be preferable.

This combined procedure begins with the laparoscopic approach, followed by the robotic approach. In cases where intraoperative evaluation prior to switching to the robotic approach indicates the need for RN, the surgery can proceed with the laparoscopic approach. In such cases, a laparoscopic radical nephrectomy offers a more practical and faster solution [18]. Another advantage is that centers with prior experience in both LPN and RAPN can adopt CPN with ease, without requiring additional training or adaptation [17]. Therefore, as the knowledge of CPN increases, this approach is expected to be more easily validated within such centers.

Our study has a few limitations. It was conducted at a single center, was retrospective in nature, and involved a single surgeon. The number of patients was not very large and also lacks patient-reported outcomes. Moreover, the study lacks long-term follow-up, which limits conclusions regarding oncological and renal outcomes. As long-term renal function data were not available, the impact of the observed technical differences on sustained renal outcomes could not be determined. While the intraoperative decision to convert from LPN to CPN was based on subjective surgeon judgment, introducing a potential bias in the comparison of perioperative outcomes, each surgical approach was performed in patients considered most appropriate; therefore, randomization could not be applied. Additionally, the trocars were changed during the procedure as part of the transition from the laparoscopic to the robotic approach. We observed that these adjustments were performed feasibly and safely, with no difficulties or complications encountered during the process. While we preferred to change the trocars, it is also possible to perform the procedure by placing 8 mm robotic trocars inside the 12 mm laparoscopic trocars, if desired.

Future prospective studies should expand upon these findings through larger multicenter cohorts and longer follow-up periods to better characterize both oncological outcomes and kidney-specific functional recovery and to validate the long-term efficacy and broader applicability of CPN. In this context, long-term renal function should be evaluated using objective functional imaging modalities such as DMSA or MAG3 renography, which may more clearly demonstrate whether the technical advantages offered by different surgical approaches are reflected in meaningful differences in functional preservation. Additionally, the transition from LPN to CPN currently relies on subjective surgeon judgment; therefore, future research should aim to develop structured and validated intraoperative decision-making criteria to minimize bias and enhance reproducibility across different surgical teams. As part of such objective criteria, the integration of intraoperative endoscopic ultrasonography or hybrid techniques that provide real-time visualization of tumor depth and parenchymal involvement may help guide conversion decisions and further improve the safety and precision of tumor excision in CPN. Moreover, the feasibility of trocar adjustments and combined laparoscopic and robotic procedural strategies should be assessed across different centers and various robotic surgery platforms. The accumulation of evidence across these domains will be essential for defining optimal surgical strategies and standardizing clinical practice in nephron-sparing surgery.

## 5. Conclusions

Our study demonstrates that combined partial nephrectomy (CPN), which incorporates laparoscopic dissection for renal mobilization followed by robotic assistance for tumor excision and intracorporeal suturing, is a feasible and safe surgical approach for clinical stage 1 renal tumors. Compared with LPN and RAPN, CPN was found to have comparable surgical margin status, early oncological outcomes, and early postoperative renal functional results, and it may offer shorter operative duration, reduced warm ischemia time, and lower estimated blood loss. This hybrid approach enables intraoperative adaptability, allowing surgeons to tailor the strategy based on tumor complexity. It also offers a practical and resource-conscious option for centers equipped with both laparoscopic and robotic platforms, without necessitating additional training. Given its low complication rates and technical advantages, CPN may represent a valuable alternative in the minimally invasive management of small renal masses. Further prospective studies are needed to validate its long-term efficacy and broader applicability.

## Figures and Tables

**Table 1:** Comparison of preoperative parameters of patients underwent laparoscopic, robot-assisted laparoscopic, and combined partial nephrectomy.

	LPN n = 31	RAPN n = 16	CPN n = 52	*p* Value
Age, years (mean ± SD)	59.6 ± 10.9	59.6 ± 8.4	59.4 ± 11.9	0.935
Gender, male, n (%)	26 (83.9)	12 (75.0)	43 (82.7)	0.735
BMI score (mean ± SD)	28.1 ± 5.0	28.0 ± 4.7	27.7 ± 4.2	0.806
Hypertension, n (%)	12 (38.7)	9 (56.3)	23 (44.2)	0.518
Previous cardiovascular disease, n (%)	13 (41.9)	8 (50.0)	15 (25.8)	0.226
Diabetes, n (%)	11 (35.5)	4 (25.0)	13 (25.0)	0.562
Previous stroke, n (%)	2 (6.5)	0 (0)	3 (5.8)	0.596
Preoperative hemoglobin (Hb) (g/dL) (mean ± SD)	14.4 ± 1.2	13.7 ± 1.0	14.4 ± 1.2	0.141
Preoperative serum creatinine (Cre) (mg/dL) (median, IQR)	0.9 (0.7–1.0)	0.9 (0.7–1.0)	0.9 (0.7–1.0)	0.884
Preoperative eGFR (mL/min/1.73 m^2^) (median, IQR)	96.6 (83.2–108.8)	85.6 (70.7–100.4)	94.7 (71.3–100.0)	0.115
Side of tumor, n (%) Right Left	16 (51.6) 15 (48.4)	7 (43.7) 9 (56.3)	24 (46.1) 28 (53.9)	0.884
Polar localization of tumor, n (%) Upper pole Middle Lower pole	12 (38.7) 10 (32.3) 9 (29.0)	6 (37.5) 6 (37.5) 4 (25.0)	19 (36.5) 18 (34.6) 15 (28.8)	0.996
Axial localization of tumor, n (%) Medial Lateral Anterior Posterior	7 (22.6) 12 (38.7) 5 (16.1) 7 (22.6)	5 (31.3) 5 (31.3) 2 (12.5) 4 (25.0)	20 (38.5) 16 (30.8) 6 (11.5) 10 (19.2)	0.873

BMI = body mass index, eGFR = estimated glomerular filtration rate, SD = Standard deviation, IQR = Interquartile range. *p* < 0.016 were considered as statistically significant.

**Table 2:** Comparison of tumor size and nephrometry scores in patients underwent laparoscopic, robot-assisted laparoscopic and combined partial nephrectomy.

	LPN n = 31	RAPN n = 16	CPN n = 52	*p* Value for CPN vs. LPN	*p* Value for CPN vs. RAPN	*p* Value for LPN vs. RAPN
Tumor size (mm) (median, IQR)	27.5 (20.0–36.7)	34.0 (31.0–41.0)	36.5 (32.2–44.7)	**0.001**	**0.005**	0.405
Preoperative RENAL nephrometry score (median, IQR)	6.0 (5.0–7.0)	6.0 (5.2–7.7)	5.0 (4.0–5.0)	**<0.001**	0.340	**0.001**
Intraoperative RENAL nephrometry score (median, IQR)	5.0 (4.0–7.0)	6.0 (4.0–9.0)	7.5 (5–9)	**<0.001**	0.03	**0.001**

RENAL = Radius, Exophytic or endophytic, Nearness to collecting system, Anterior or posterior, Location, IQR = Interquartile range. *p* < 0.016 were considered as statistically significant. Statistically significant *p* values are given in bold.

**Table 3:** Comparison of tumor size and nephrometry scores in patients underwent laparoscopic, robot-assisted laparoscopic and combined partial nephrectomy after PSM.

	CPN n = 31	LPN n = 31	*p* Value for CPN vs. LPN	CPN n = 16	RAPN n = 16	*p* Value for CPN vs. RaPN	LPN n = 16	RAPN n = 16	*p* Value for LPN vs. RaPN
	Group 1	Group 2		Group 1	Group 3		Group 2	Group 3	
Tumor size (mm) (median, IQR)	35.0 (28.0–42.0)	34.0 (31.0–39.5)	0.544	42.0 (31.0–46.5)	36.5 (32.5–44.5)	0.515	36.5 (29.0–49.0)	36.5 (32.5–44.5)	0.956
Preoperative RENAL nephrometry score (median, IQR)	7.0 (5.0–7.0)	5.0 (4.0–5.0)	**<0.001**	7.0 (7.0–8.0)	7.5 (6.5–8.0)	0.468	4.5 (4.0–6.0)	7.5 (6.5–8.0)	**<0.001**

RENAL = Radius, Exophytic or endophytic, Nearness to collecting system, Anterior or posterior, Location, IQR = Interquartile range, PSM = propensity score matching. Statistically analyzed with Mann–Whitney U test. *p* < 0.05 were considered as statistically significant. Statistically significant *p* values are given in bold.

**Table 4:** Comparison of perioperative parameters of patients undergoing laparoscopic, robot-assisted laparoscopic, and combined partial nephrectomy.

	LPN n = 31	RAPN n = 16	CPN n = 52	*p* Value for CPN vs. LPN	*p* Value for CPN vs. RAPN	*p* Value for LPN vs. RAPN
Kidney dissection time (min) (median, IQR)	45.7 (36.2–62.3)	97.5 (87.7–106.5)	48.0 (43.0–53.0)	0.480	**<0.001**	**<0.001**
Tumor resection time (min) (median, IQR)	8.0 (4.0–12.0)	5 (3.0–8.0)	6.0 (3.0–11.5)	**0.012**	0.023	**<0.001**
Parenchymal reconstruction time (min) (median, IQR)	16.0 (11.0–23.0)	13.5 (10.0–16.0)	13.0 (6.0–20.0)	**<0.001**	0.500	**0.001**
Warm ischemia time (WIT) (min) (median, IQR)	23.0 (18.0–28.0)	18.5 (14.0–23.0)	20.0 (10.0–26.0)	**<0.001**	0.158	**<0.001**
Estimated blood loss (EBL) (mL) (median, IQR)	180.0 (100.0–250.0)	110.0 (50.0–300.0)	120.0 (50.0–350.0)	**0.003**	0.416	**0.003**
Total operative time (OT) (min) (mean ± SD)	136.87 ± 9.85	155.5 ± 18.03	126.75 ± 25.28	**0.014**	**<0.001**	**<0.001**

SD = Standard deviation, IQR = Interquartile range. *p* < 0.016 were considered as statistically significant. Statistically significant *p* values are given in bold.

**Table 5:** Comparison of perioperative parameters of patients undergoing laparoscopic, robot-assisted lap-aroscopic, and combined partial nephrectomy after PSM.

	CPN n = 31	LPN n = 31	*p* Value for CPN vs. LPN	CPN n = 16	RAPN n = 16	*p* Value for CPN vs. RaPN	LPN n = 16	RAPN n = 16	*p* Value for LPN vs. RaPN
	Group 1	Group 2		Group 1	Group 3		Group 2	Group 3	
Kidney dissection time (min) (mean ± SD)	49.4 ± 18.6	48.8 ± 7.1	0.875	48.5 ± 16.5	97.4 ± 13.1	**<0.001**	49.8 ± 7.2	97.4 ± 13.1	**<0.001**
Tumor resection time (min) (mean ± SD)	6.3 ± 2.1	7.4 ± 1.9	**0.032**	6.2 ± 2.0	5.0 ± 1.5	0.059	7.1 ± 2.1	5.0 ± 1.5	**0.003**
Parenchymal reconstruction time (min) (median, IQR)	13.0 (10.0–14.0)	16.0 (14.5–16.7)	**<0.001**	11.7 (10.0–14.75)	13.5 (11.5–14.5)	0.341	16.0 (15.0–16.25)	13.5 (11.5–14.5)	**<0.001**
Warm ischemia time (WIT) (min) (median, IQR)	19.0 (17.0–21.7)	23.0 (21.2–25.0)	**<0.001**	21.2 (16.5–22.2)	18.5 (15.5–21.0)	0.287	22.5 (21.0–25.5)	18.5 (15.5–21.0)	**<0.001**
Estimated blood loss (EBL) (mL) (mean ± SD)	141.7 ± 70.0	176.2 ± 47.2	**0.027**	148.4 ± 71.2	129.0 ± 62.1	0.419	164.3 ± 48.0	129.0 ± 62.1	0.082
Total operative time (OT) (min) (median, IQR)	126 (109.0–138.5)	134.0 (130.0–138.0)	**0.029**	132.5 (117.0–140.5)	152.5 (145.5–166.5)	**0.001**	134.5 (130.0–136.0)	152.5 (145.5–166.5)	**<0.001**

SD = Standard deviation, IQR = Interquartile range, PSM = propensity score matching. Statistically analyzed with Student’s t-test or Mann–Whitney U test depends on normality. *p* < 0.05 were considered as statistically significant. Statistically significant *p* values are given in bold.

**Table 6:** Comparison of postoperative parameters of patients undergoing laparoscopic, robot-assisted laparoscopic, and combined partial nephrectomy.

	LPN n = 31	RAPN n = 16	CPN n = 52	*p* Value
Hemoglobin decrease (g/dL) (median, IQR)	1.6 (1.0–1.8)	1.3 (1.2–1.5)	1.5 (1.1–2.3)	0.447
Serum creatinine increase (mg/dL) (median, IQR)	0.3 (0.2–0.4)	0.2 (0.2–0.2)	0.2 (0.1–0.4)	**0.013**
eGFR decrease (mL/min/1.73 m^2^) (mean ± SD)	24.1 ± 9.8	16.2 ± 6.4	18.8 ± 13.6	0.156
Hospitalization time (days) (median, IQR)	3.0 (3.0–4.0)	3.0 (2.0–3.7)	3.0 (2.0–4.0)	0.228
Complications, n (%) Clavien-Dindo grade 1 Clavien-Dindo grade 2 Clavien-Dindo grade 3 Clavien-Dindo grade 4	2 (6.4) 2 (6.4) 0 (0) 0 (0)	1 (6.2) 1 (6.2) 0 (0) 0 (0)	2 (3.8) 3 (5.7) 0 (0) 0 (0)	1.000
Histology, n (%) Clear cell RCC Papillary RCC Oncocytoma Chromophobe RCC Angiomyolipoma Undifferentiated RCC Benign renal cyst Oncocytic carcinoma Sarcoma	14 (45.2) 7 (22.6) 3 (9.7) 2 (6.5) 3 (9.7) 2 (6.5) 0 (0) 0 (0) 0 (0)	9 (56.3) 3 (18.8) 1 (6.3) 1 (6.3) 2 (12.5) 0 0 0 0	29 (55.8) 6 (11.5) 5 (9.6) 5 (9.6) 3 (5.8) 1 (1.9) 1 (1.9) 1 (1.9) 1 (1.9)	0.951

eGFR = estimated glomerular filtration rate, RCC = Renal Cell Carcinoma,,SD = Standard deviation, IQR = Interquartile range. *p* < 0.016 were considered as statistically significant. Statistically significant *p* values are given in bold, p^a^ comparison of CPN and LPN groups, p^b^ comparison of CPN and RAPN, p^c^ comparison of LPN and RAPN. For Serum creatinine increase; p^a^ = 0.046, p^b^ = 0.524, **p^c^= 0.001**.

## Data Availability

The findings generated and/or analyzed in this study are available from the corresponding author upon reasonable request.

## References

[1] Bray F., Laversanne M., Sung H., Ferlay J., Siegel R.L., Soerjomataram I., Jemal A. (2024). Global cancer statistics 2022: GLOBOCAN estimates of incidence and mortality worldwide for 36 cancers in 185 countries. CA A Cancer J. Clin..

[2] Capitanio U., Bensalah K., Bex A., Boorjian S.A., Bray F., Coleman J., Gore J.L., Sun M., Wood C., Russo P. (2019). Epidemiology of Renal Cell Carcinoma. Eur. Urol..

[3] Okhawere K.E., Pandav K., Grauer R., Wilson M.P., Saini I., Korn T.G., Meilika K.N., Badani K.K. (2023). Trends in the surgical management of kidney cancer by tumor stage, treatment modality, facility type, and location. J. Robot. Surg..

[4] Zabell J.R., Wu J., Suk-Ouichai C., Campbell S.C. (2017). Renal Ischemia and Functional Outcomes Following Partial Nephrectomy. Urol. Clin. N. Am..

[5] Calpin G.G., Ryan F.R., McHugh F.T., McGuire B.B. (2023). Comparing the outcomes of open, laparoscopic and robot-assisted partial nephrectomy: A network meta-analysis. BJU Int..

[6] Wexner S.D., Bergamaschi R., Lacy A., Udo J., Brölmann H., Kennedy R.H., John H. (2009). The current status of robotic pelvic surgery: Results of a multinational interdisciplinary consensus conference. Surg. Endosc..

[7] Ruiz Guerrero E., Claro A.V.O., Ledo Cepero M.J., Soto Delgado M., Álvarez-Ossorio Fernández J.L. (2023). Robotic versus laparoscopic partial nephrectomy in the new era: Systematic review. Cancers.

[8] DeLong J.M., Shapiro O., Moinzadeh A. (2010). Comparison of laparoscopic versus robotic assisted partial nephrectomy: One surgeon’s initial experience. Can. J. Urol..

[9] Thakker P.U., O’Rourke T.K., Hemal A.K. (2023). Technologic advances in robot-assisted nephron sparing surgery: A narrative review. Transl. Androl. Urol..

[10] Kutikov A., Uzzo R.G. (2009). The RENAL nephrometry score: A comprehensive standardized system for quantitating renal tumor size, location and depth. J. Urol..

[11] Dindo D., Demartines N., Clavien P.-A. (2004). Classification of Surgical Complications: A New Proposal With Evaluation in a Cohort of 6336 Patients and Results of a Survey. Ann. Surg..

[12] Amin M.B., Greene F.L., Edge S.B., Compton C.C., Gershenwald J.E., Brookland R.K., Meyer L., Gress D.M., Byrd D.R., Winchester D.P. (2017). The eighth edition AJCC cancer staging manual: Continuing to build a bridge from a population-based to a more “personalized” approach to cancer staging. CA A Cancer J. Clin..

[13] Tunc L., Canda A.E., Polat F., Onaran M., Atkin S., Biri H., Bozkirli I. (2011). Direct upper kidney pole access and early ligation of renal pedicle significantly facilitates transperitoneal laparoscopic nephrectomy procedures: Tunc technique. Surg. Laparosc. Endosc. Percutan. Tech..

[14] Lucas S.M., Mellon M.J., Erntsberger L., Sundaram C.P. (2012). A comparison of robotic, laparoscopic and open partial nephrectomy. JSLS J. Soc. Laparoendosc. Surg..

[15] Hinata N., Shiroki R., Tanabe K., Eto M., Takenaka A., Kawakita M., Hara I., Hongo F., Ibuki N., Nasu Y. (2021). Robot-assisted partial nephrectomy versus standard laparoscopic partial nephrectomy for renal hilar tumor: A prospective multi-institutional study. Int. J. Urol..

[16] Ginsburg K.B., Schober J.P., Kutikov A. (2022). Ischemia Time Has Little Influence on Renal Function Following Partial Nephrectomy: Is It Time for Urology to Stop the Tick-Tock Dance?. Eur. Urol..

[17] Alimi Q., Peyronnet B., Sebe P., Cote J.-F., Kammerer-Jacquet S.-F., Khene Z.-E., Pradere B., Mathieu R., Verhoest G., Guillonneau B. (2018). Comparison of short-term functional, oncological, and perioperative outcomes between laparoscopic and robotic partial nephrectomy beyond the learning curve. J. Laparoendosc. Adv. Surg. Tech..

[18] Asimakopoulos A.D., Miano R., Annino F., Micali S., Spera E., Iorio B., Vespasiani G., Gaston R. (2014). Robotic radical nephrectomy for renal cell carcinoma: A systematic review. BMC Urol..

[19] Aboumarzouk O.M., Stein R.J., Eyraud R., Haber G.-P., Chlosta P.L., Somani B.K., Kaouk J.H. (2012). Robotic versus laparoscopic partial nephrectomy: A systematic review and meta-analysis. Eur. Urol..

[20] Routh J.C., Bacon D.R., Leibovich B.C., Zincke H., Blute M.L., Frank I. (2008). How long is too long? The effect of the duration of anaesthesia on the incidence of non-urological complications after surgery. BJU Int..

[21] Artsitas S., Artsitas D., Koronaki I., Toutouzas K.G., Zografos G.C. (2024). Comparing robotic and open partial nephrectomy under the prism of surgical precision: A meta-analysis of the average blood loss rate as a novel variable. J. Robot. Surg..

[22] Sri D., Thakkar R., Patel H., Lazarus J., Berger F., McArthur R., Lavigueur-Blouin H., Afshar M., Fraser-Taylor C., Le Roux P. (2021). Robotic-assisted partial nephrectomy (RAPN) and standardization of outcome reporting: A prospective, observational study on reaching the “Trifecta and Pentafecta”. J. Robot. Surg..

[23] Malthouse T., Kasivisvanathan V., Raison N., Lam W., Challacombe B. (2016). The future of partial nephrectomy. Int. J. Surg..

